# High Performance Seesaw Torsional CMOS-MEMS Relay Using Tungsten VIA Layer

**DOI:** 10.3390/mi9110579

**Published:** 2018-11-07

**Authors:** Martín Riverola, Francesc Torres, Arantxa Uranga, Núria Barniol

**Affiliations:** Department of Electronics Engineering, Universitat Autònoma de Barcelona, 08193 Bellaterra, Spain; Martin.riverola@gmail.com (M.R.); francesc.torres@uab.es (F.T.); arantxa.uranga@uab.es (A.U.)

**Keywords:** MEMS relays, MEMS switches, mechanical relays, CMOS-MEMS, MEMS

## Abstract

In this paper, a seesaw torsional relay monolithically integrated in a standard 0.35 μm complementary metal oxide semiconductor (CMOS) technology is presented. The seesaw relay is fabricated using the Back-End-Of-Line (BEOL) layers available, specifically using the tungsten VIA3 layer of a 0.35 μm CMOS technology. Three different contact materials are studied to discriminate which is the most adequate as a mechanical relay. The robustness of the relay is proved, and its main characteristics as a relay for the three different contact interfaces are provided. The seesaw relay is capable of a double hysteretic switching cycle, providing compactness for mechanical logic processing. The low contact resistance achieved with the TiN/W mechanical contact with high cycling life time is competitive in comparison with the state-of-the art.

## 1. Introduction

It is expected that new micro- and nanoelectromechanical (M/NEM) relays can play an important role as a new device for adding functionality and decreasing the power consumption for the more demanding area of consumable devices (IoT, wearables) [1]. One of the important things in mechanical relays is the capability of a quasi-ideal switching behavior (with a very abrupt on-off switching, and zero current leakage during the OFF-state) and multi-terminal operation which can serve to save energy, as it has been envisioned in several different digital applications [2,3,4,5]. The possibility of using the complementary metal oxide semiconductor (CMOS) platform for the monolithic fabrication of such M/NEMS relays in a real combination with classical CMOS devices can open a myriad of new possibilities for decreasing power consumption. Additionally, the high number of metal layers used in the advanced CMOS technology nodes make very attractive the exploitation of a CMOS-MEMS platform for using metal layers, not only as an electrical connection path, but also to provide some active processing using these layers as embedded MEMS devices [6,7]. Despite this interest in obtaining functional mechanical switching devices embedded in CMOS, most of the presented examples from the literature are only CMOS-compatible [8,9,10,11,12], with few of them being really embedded in CMOS [13,14,15,16,17]. In all cases, the devices are far from possessing all of the ideal characteristics (low contact resistance, low operation voltage and high yield). For instance, the TiN coated relay presented in [8] presents a non-ohmic contact resistance with a high life cycling, while the similarly TiN coated PolySilicon relay in [9] has low contact resistance, but presents limited cycling operation. In Reference [10], a NEMS relay with a very low pull-in voltage (0.4 V) is presented, but it is only operable for 20 cycles. In Reference [11], a demonstration of a CMOS driven Pt-NEMS relay fabricated over the CMOS is presented, but with a very high contact resistance (100 MΩ) and without testing the life time of the relay. Reference [12] presents a two-terminal TiN NEMS relay fabricated under a CMOS compatible process with an operability of hundreds of cycles, but with a limited current operation (nA range). Concerning papers with MEMS relays embedded in CMOS, similar problems are encountered. Papers using the same CMOS-MEMS tungsten-based relay as presented in this paper, but with different configurations and designs, suffers from these non-ideal characteristics: Reference [13] presents a torsional relay with a high pull-in voltage and below one hundred operation cycling; References [14,15] are based on lateral relays exhibiting in both cases a high contact resistance (1 MΩ and 750 MΩ in References [14,15], respectively). Even higher contact resistances and low cycling operation are encountered in other CMOS-MEMS approaches: In Reference [16], contact resistance is greater than 500 MΩ and 30 operation cycles; in Reference [17], the contact resistance is in the GΩ range and only 10 operation cycles. As a consequence of these reported characteristics, more research is necessary in order to improve the performance of these CMOS-MEMS relays.

In this paper, we present new MEMS devices capable of providing five-terminal relays with a bidirectional operation and embedded in CMOS, demonstrating enhanced performance compared with the already reported TiN-based MEMS relays. The main issue with the fabrication of the presented relays is the use of the tungsten VIA of the conventional AMS (Austria Microsystems) 0.35 μm CMOS technology. The exploitation of the VIA3 made from tungsten as the main structural layer for CMOS-MEMS devices presents a series of attractive characteristics that are suitable for mechanical relays: high hardness, being resistant to wear and plastic deformation; high melting point (tungsten exhibits the highest melting point); being resistant to welding-induced failure due to Joule heating at the contact. Furthermore, VIA3 is a top Back-End-Of-Line (BEOL) layer more thinly covered in SiO_2_, which implies small releasing times, and thus increased yield in the fabrication process.

The use of the tungsten VIA3 has been demonstrated previously for MEMS devices: resonators for monolithically CMOS-MEMS stand-alone oscillators [18,19], relays for switching applications [13,14,15], and very recently as CO_2_ transducers [20]. All these applications demonstrate the importance of the approach and the opportunity to explore new MEMS structures and devices based on this tungsten VIA3 approach. In this paper we will focus on a mechanical five-terminal relay working in its torsional operation with an enhancement of the electrostatic coupling, and consequently lower pull-in voltage, and a decrease of the contact resistance due to the ability to define larger contact areas compared with the above reported examples. Moreover, the paper studies all the different contact materials available in the BEOL-CMOS metal layers without adding any additional metallization in order to provide a totally monolithic integration with CMOS. From the presented results we can state that the CMOS-MEMS relay with TiN-W contacts presents the highest ON-OFF current ratio (10^7^), the lowest contact resistance 2 kΩ, and the highest cycling life test compared with the state-of-the-art MEMS relays based on TiN contacts [8,9,12,13,14,15,16,17]. 

## 2. Materials and Methods

### 2.1. Device Design and Fabrication

**Figure 1:**
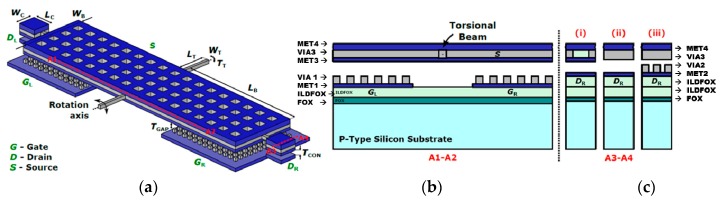
(**a**) 3D schematic of the designed seesaw relay including cross-sectional views (red lines). (**b**) Cross-section A1–A2 along the length of the relay, the gate electrodes are defined with MET1 and VIA1. (**c**) Cross-section A3–A4 at the contact area of the relay (between Source and Drain) with the three possibilities: (i) Type I, (ii) Type II, and (iii) Type III.

The torsional relay consists of a five-terminal seesaw device schematically drawn in Figure 1. The seesaw relay design consists in a main plate formed by two sandwiched metal layers (MET3 and MET4) of the CMOS technology contacted through the contacting metal VIA layer (specifically, a sandwiched MET4-VIA3-MET3). This main plate is anchored by two VIA3 torsional beams (called source, S) which allow the ends of the main beam to move up and down by electrostatically actuating the relay with the basally located gate electrodes (G_R_ and G_L_). This gate electrode is formed by metal layer (MET1) and its contacting VIA (VIA1). Three types of endings (the final contacts for the seesaw relays) are made (see cross-section A3–A4 in Figure 1c): (a) Type I, MET4-VIA3-MET3, which make contact with the drain electrodes made by MET2, (b) Type II, MET4-VIA3, which make contact with the drain electrodes of MET2; and (c) Type III, MET4-VIA3, which makes contact with the drain electrodes defined in this case with MET2-VIA2. Each of the metal layers (METi) of the 0.35 μm CMOS technology are a sandwiched layer consisting of TiN/Al/TiN. In this sense, three kinds of contacts will be characterized: (a) TiN vs. TiN in type I relays; (b) W vs. TiN in type II relays; and (c) W vs. W in type III relays. Note that these three types of relays will provide contact gaps at different heights.

The design parameter values for the three types of relays are listed in Table 1. The parameters used have been chosen taking into account the following requirements: (a) torsional actuation selecting VIA3 torsional beams to have an equivalent torsional spring constant smaller than the vertical actuation, using the minima dimension for the VIA3 width (*W_T_* = 0.5 μm), and gate electrodes (G_R_ and G_L_) are situated at the end of the body to promote torsional movement; (b) maximize actuation area (gate electrodes size and body size) between MET3 and MET1 to minimize pull-in voltage in comparison with previous designs [13] (note that the VIA1 contacts used over the MET1 are intended to enhance electrostatic coupling between actuation electrodes and relay body to further reduce pull-in voltage); (c) squared contact area of 2.5 µm × 2.5 µm to decrease contact resistance. All of the other parameters are constraints from the CMOS technology used. Due to the non-uniform material based seesaw relay, as well as to the structure of the gate electrodes (with the small metal contacts, VIA1), it is not possible to analytically compute the behavior of the seesaw relay (i.e., pull-in voltage). Consequently, finite-element-model simulations using Coventor have been extensively used to tune design parameters (Table 2 summarizes the main simulated characteristics for the seesaw relays).

The fabrication process of the VIA3 MEMS structures is based on a mask-less wet-etching process [21,22]. A passivation aperture is defined over the resonator which allows this in-house post-CMOS MEMS releasing process to be done directly while the passivation layer of silicon nitride is used as a protective layer for the rest of the chip. The releasing process consists basically of three steps: (a) isotropic wet-etching in a bath of buffered hydrofluoric acid solution at room temperature (with an oxide etching rate of around 300 nm/min [21]); (b) chip washing in distilled water followed by an isopropyl alcohol bath to eliminate the water; and (c) heating in an oven at 100 °C to evaporate the remaining alcohol. No sticking problems have been encountered for the seesaw MEMS relay with this etching, which does not require critical point drying for the releasing. As it is an isotropic process, the etching time depends on the MEMS dimensions and the quantity of oxide over the structure. In the case of the seesaw relays, and due to the large area of the body structure, releasing holes have been included to facilitate the wet etching of the sacrificial SiO_2_ layer underneath the large main plate. The etching time used was typically in the range between 10 and 18 min. This etching process is CMOS compatible, as it has already been demonstrated with VIA3 MEMS structures embedded in functional CMOS circuitry [18,19,23]. 

It is necessary to ensure that the torsional mode operation of the seesaw relay dominates over the flexural mode operation while it is switching. Therefore, the vertical flexural spring constant must be much stiffer than the torsional spring constant. Table 2 shows the simulated resonant frequency of the torsional and vertical mode and their respective effective stiffness using the following material properties: Young modulus of 410 GPa, 70 GPa and 600 GPa, and mass densities of 19,300 kg/m^3^, 2700 kg/m^3^ and 5430 kg/m^3^ for tungsten, aluminum and titanium nitride, respectively. As can be seen, the vertical spring constant is 55× higher than the torsional spring constant.

**Figure 2:**
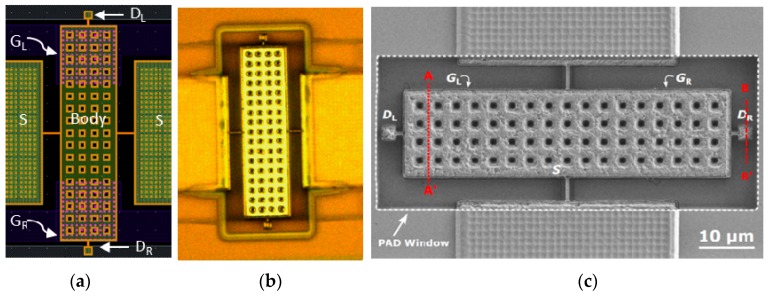

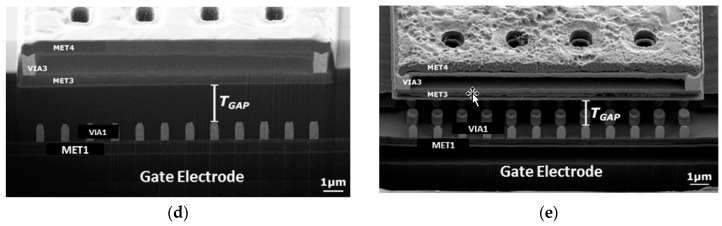
(**a**) Layout of the seesaw relay. The gate electrodes are the pink areas below the body structure. (**b**) Top view optical image of fabricated and released seesaw relay. (**c**) Top view SEM image indicating the cut-line A-A′ over the body structure and B-B’ over the contact area. (**d**,**e**) SEM images of the cross-section in the A-A′ cut-line (**d**) before and (**e**) after the releasing process. These images allow one to see the gate electrodes composed by the MET1 and VIA1 layers, as well as the sandwiched composition of the body element of the relay (a sandwich of MET3-VIA3-MET4).

**Figure 3:**
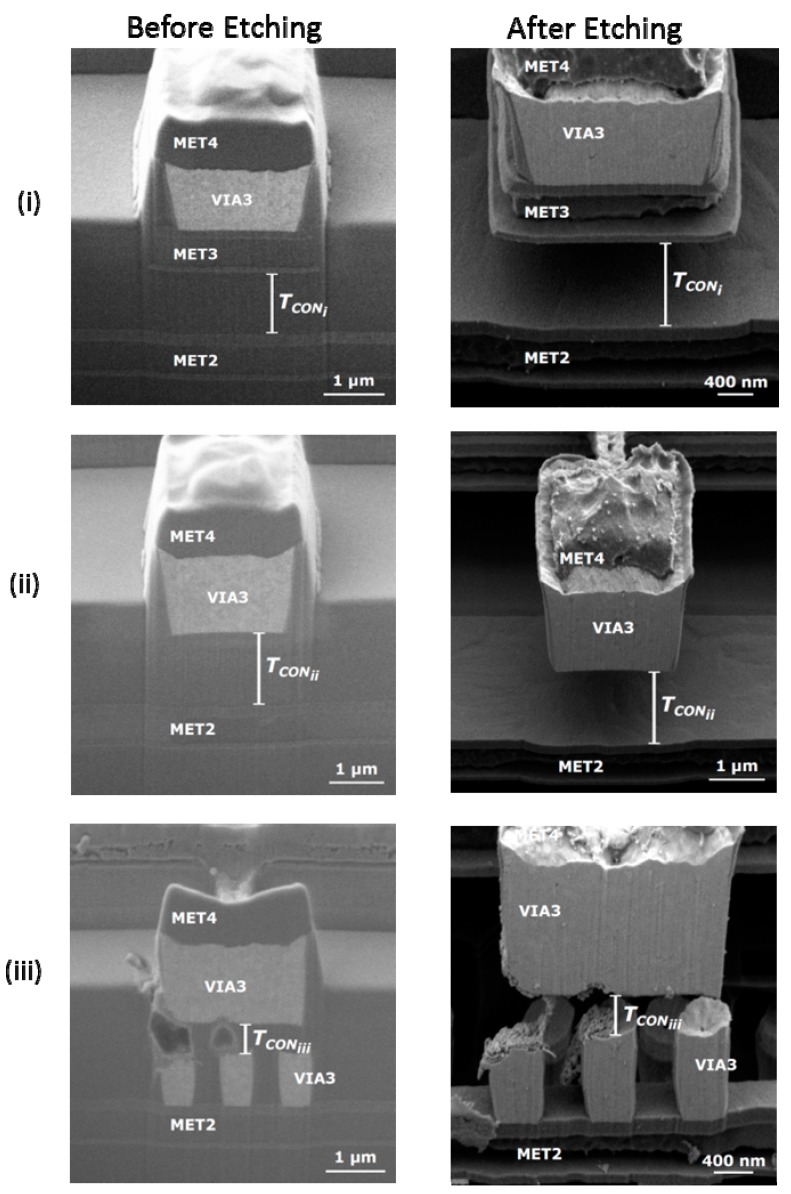
SEM images of the cross-section B-B’ in Figure 2c over the contact in the three different designs (contact between body relay with different composition and drain) showing before (left images) and after (right images) the releasing: (**i**) Type I, (**ii**) Type II and (**iii**) Type III. These figures can be compared with the cross-section A3-A4 at the contact area of the relay in Figure 1, in which the different composition for contact source and drain are explained.

Figure 2 and Figure 3 show the layout, optical and SEM images of the fabricated seesaw relays, along with the focused ion beam (FIB) cross-sectional views to detail the different technological implementations of the relay body (Figure 2) and relay contact (Figure 3). The cross-sections are provided before and after the releasing of the seesaw relay. From these images, the gap distances of the relay contact (Table 1) are extracted.

### 2.2. Electrical Characterization

**Figure 4:**
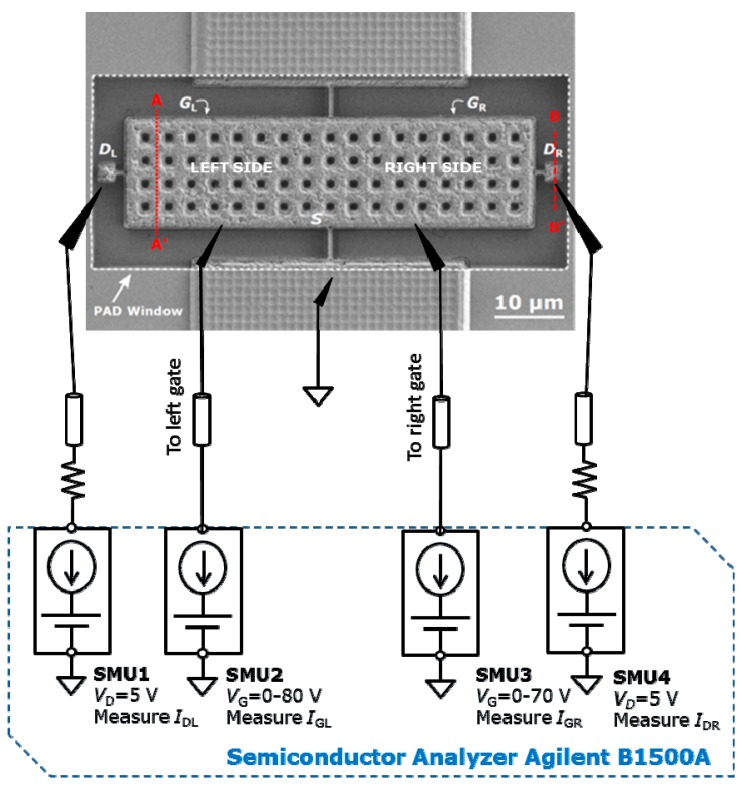
Electrical set-up for the current voltage (*I-V*) switching characteristics of the five-terminal relay. Four high-resolution source measure units (SMU) are used: the source electrode (relay structure) is grounded, drain electrodes (left and right) are fixed to a VD = 5 V, gate electrodes (left and right) are swept up and down from 0 to a voltage gate V_G_ voltage higher than the pull-in voltage, V_PI_. Note that gates and drains are underneath the relay structure and are not visible in the image.

The fabricated seesaw relays were tested under two different conditions: (1) at room temperature in air at atmospheric pressure, and (2) under vacuum at 10^−5^ mbar. In ambient conditions, the chips were exposed to air and tested in a Cascade Microtech probe station (PM8). Under vacuum conditions, the chip was mounted and bounded onto a printed circuit boardand placed inside a homemade vacuum chamber. The current-voltage (*I-V*) characterization was performed with an Agilent semiconductor analyzer B1500A equipped with four high-resolution source measure units (SMU) (Figure 4).

## 3. Results

In this section, the current voltage (*I-V*) curves for the three types of fabricated seesaw relays placed in both air conditions and vacuum conditions are reported. The pull-in and pull-out voltages, I_ON_-I_OFF_ ratio, contact resistance, and the cycling, or life-time, of the different relays are provided.

### 3.1. Seesaw Relay with Contact Type I: TiN vs. TiN

**Figure 5:**
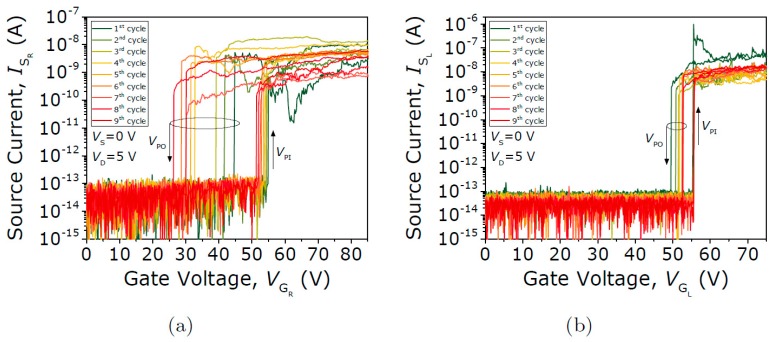
First nine current voltage (*I-V*) switching characteristics in ambient conditions of the (**a**) left and (**b**) right drain electrodes for the seesaw relay Type I (TiN-TiN contact).

**Figure 6:**
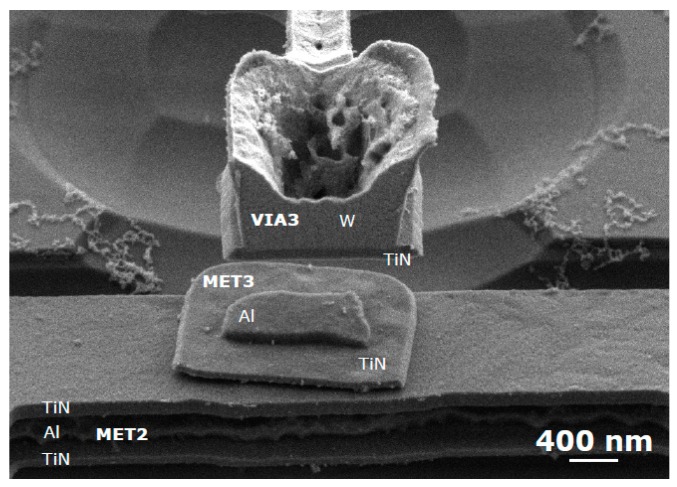
SEM image taken in the contact region of the seesaw relay showing the over-etch of the Al layer contained in the sandwiched MET3 layer of TiN-Al-TiN.

Figure 5a,b shows the first nine current voltage (*I-V*) curves taken from both the left and right ends of a seesaw relay being exposed to air conditions. As the right gate voltage V_GR_ is increased from 0 to 85 V, the right side of the torsion beam turns on abruptly at 54.8 V, while the left side remains off. Thus, a conductive path is formed between the right contact electrode (or right drain) and the movable structure (or source) by fixing the drain-to-source voltage (V_DS_) to 5 V. Similarly, the left side of the relay is also actuated by sweeping up and down the left gate voltage V_GL_ from 0 to 85 V and fixing the left drain voltage V_DL_ also to 5 V (protected with 1 MΩ). In this case, the left side turns on abruptly at 55.5 V. For both sweeps, the measured on-off current ratio is ~10^5^, and the contact resistance R_c_ is ~10^8^. Instead, an asymmetric behavior is observed comparing the V_PO_ of both tested sides. Since the V_PO_, and thus the hysteresis window, is strongly related with the adhesion forces at the contact interface, this would mean that different contact scenarios are involved in both contact ends. SEM images were taken to confirm this hypothesis, as shown in Figure 6. As can be seen, the bottom thin TiN layer that forms the sandwiched MET3 layer of TiN-Al-TiN fell over the MET2 layer due to the long wet-etching to release the structure, causing the observed asymmetry in the hysteresis window.

### 3.2. Seesaw Relay with Contact Type II: W vs. TiN

**Figure 7:**
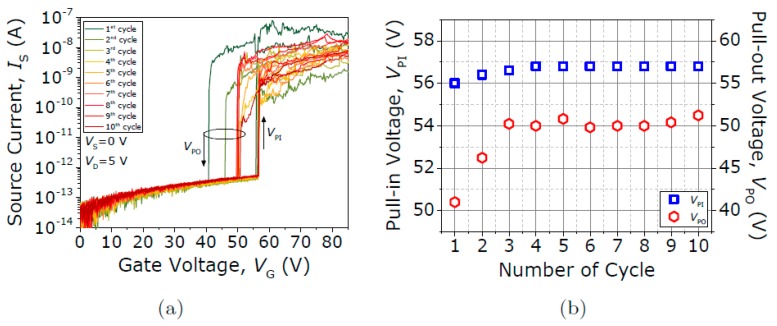
(**a**) First ten current voltage (*I-V*) switching characteristics. (**b**) Evolution of V_PI_ and V_PO_ over these ten cycles. Measures correspond to the seesaw relay Type II (W-TiN contact) in ambient conditions.

**Figure 8:**
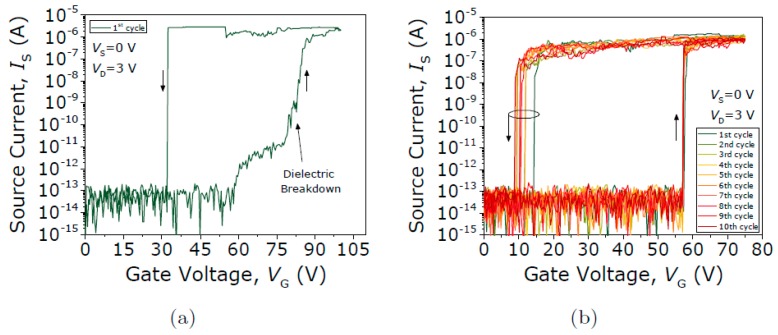
(**a**) First taken current voltage (*I-V*) curve showing no abrupt transition during the pull-in until the breakdown of the native oxide. (**b**) Next 10 current voltage (*I-V*) curves. Measures correspond to the seesaw relay Type II (W-TiN contact) under vacuum conditions.

Figure 7a shows the first ten current voltage (*I-V*) curves taken in a contact-type-(ii) seesaw relay being exposed to air conditions, exhibiting a similar Rc of ~10^8^ and an I_ON_/I_OFF_ ratio of 10^4^. Figure 7b shows how V_PI_ and V_PO_ evolve over these ten cycles. V_PI_ is fairly stable, but V_PO_ increases gradually with exposure to air. This phenomenon can be explained by the reduced surface adhesive force from metallic surfaces to oxide surfaces. Therefore, the hysteresis window reduces over time due to oxide formation in the W surface. Figure 8 shows the I-V characterization conducted under vacuum conditions at 10^−4^ mbar. The first current voltage (*I-V*) curve shows no abrupt transition due to the breakdown of the native oxide at the TiN/W contact interface (see Figure 8a). Next, ten current voltage (*I-V*) curves are taken as shown in Figure 8b, which already show the typical hysteretic behavior with initial sharp V_PI_ and V_PO_ voltages of 57.4 V and 14.6 V, respectively. The R_C_ is ~1 MΩ, 500× better compared to air conditions, which leads to an increased I_ON_/I_OFF_ ratio of 10^7^. Recall that a wider hysteresis window means that adhesion forces are exacerbated in the contacting region due to an increased effective contact area from the larger levels of current obtained.

**Figure 9:**
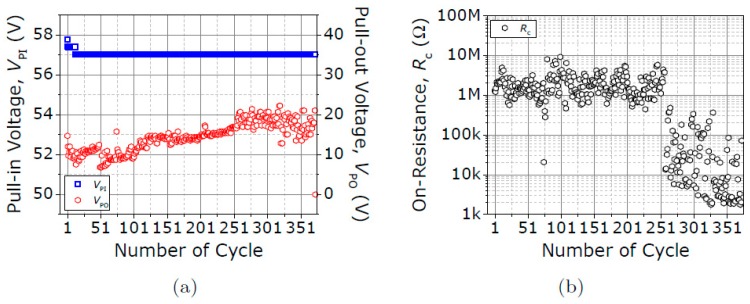
Evolution of (**a**) V_PI_, V_PO_ and (**b**) R_c_ over 355 switching cycles under vacuum conditions and V_DS_ fixed at 3 V. Compliance current set over the measured current level.

Figure 9 shows how V_PI_, V_PO_ and R_c_ evolve over a total of 355 switching cycles. Compliance was set over the maximum level of measured current. A nominal V_PI_ of 57 V is found to be stable over these cycles, with an absolute error of only 0.75 V. V_PO_ appears to increase over these cycles. Unexpectedly, it was found that R_c_ drops to 2 kΩ from cycle 251, ultimately leading to permanent stiction. This effect can be due to excessive localized Joule heating at the contact asperities, which at sufficient contact temperature, annealing of the contact takes place, reducing the contact hardness. The final 2 kΩ contact resistance is the smallest R_C_ found.

**Figure 10:**
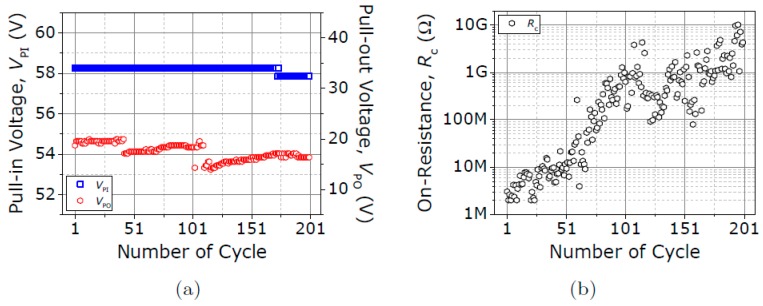
Evolution of (**a**) V_PI_, V_PO_ and (**b**) R_c_ over 200 switching cycles under vacuum conditions and V_DS_ fixed at 3 V. Compliance current limit is fixed to 1 µA.

**Figure 11:**
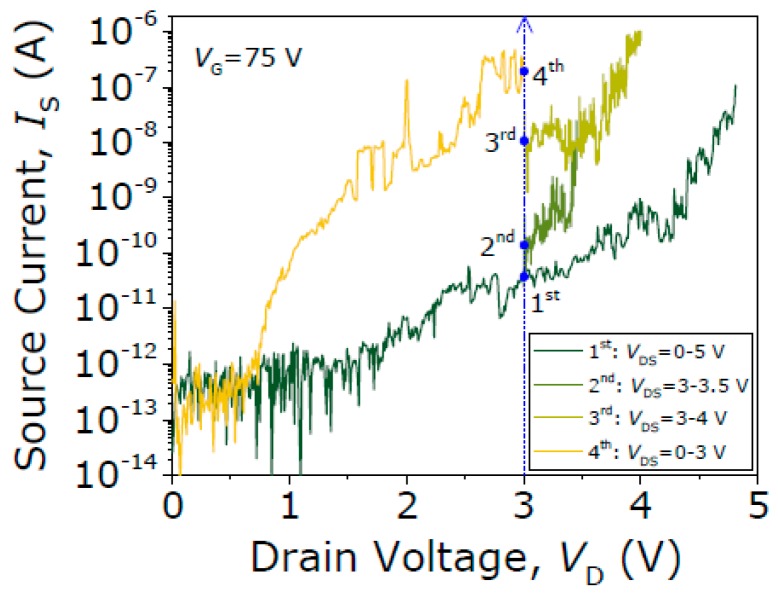
Successive source current versus drain voltage (*I_DS_-V_DS_*) sweeps showing the restoring of the contact performance after breaking down the grown native oxide on the W contact site of the relay. V_GS_ is fixed to 75 V to keep the relay in the ON-state.

The V_PI_, V_PO_ and R_c_ are recorded over 200 cycles in a new fresh relay (Figure 10), but this time keeping the compliance limit to 1 µA to avoid excessive Joule heating. The V_PI_ shows a nominal value of 58.2 V, with an absolute error of only 0.4 V over these cycles, demonstrating again the great stability of the VIA3 platform. Regarding the R_c_, it is shown to increase with the switching cycles. Therefore, the compliance limit at 1 mA favors avoiding excessive Joule heating, but favors the insulating native-oxide formation at the contacting interface (W site of the relay), increasing the R_c_. To substantiate this, Figure 11 shows the acquired current with the relay in the ON-state (V_G_ = 75 V >> V_PI_), applying higher V_DS_ voltages (V_DS_ > 3 V); the current level is higher for higher V_DS_ after breaking down the grown oxide, restoring the contact performance. This indicates that the contact endurance is not intrinsically degraded but strongly affected by the oxide regrowth.

### 3.3. Seesaw Relay with Contact Type III: W vs. W

**Figure 12:**
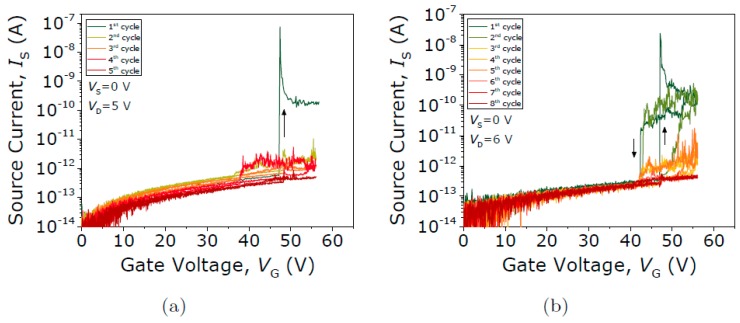
Successive current voltage (*I-V*) curves in air from the (**a**) left contact and (**b**) right contact of the Type III seesaw relay.

Figure 12 shows the I-V characterization of both left and right ends of a contact Type III seesaw relay being exposed to air conditions. It can be observed an initial symmetric V_PI_ of 47.4 and 47.1 V in the left and right ends respectively. However, the current degrades to the noise level in only five cycles. Thus, contact Type III seesaw involving W-to-W interfaces exhibit the most exacerbated degradation when cycling in air.

**Figure 13:**
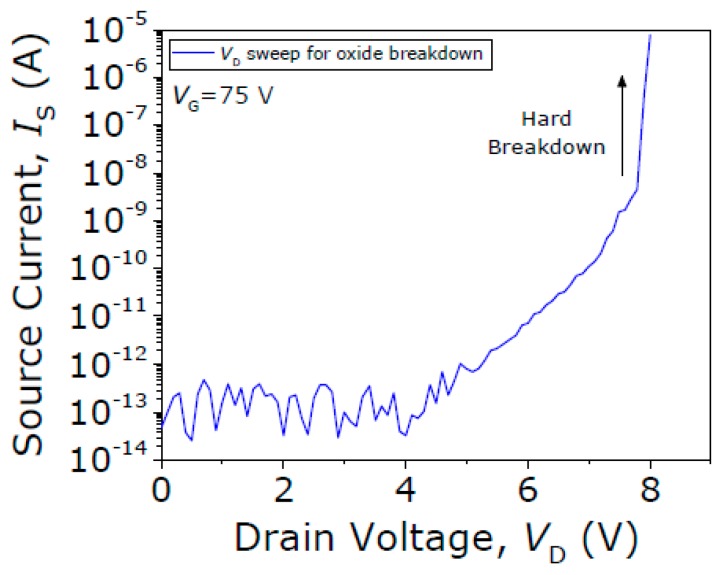
Current versus drain voltage curve in pull-in conditions to produce the initial native oxide breakdown (roughly at V_DS_ = 8 V).

**Figure 14:**
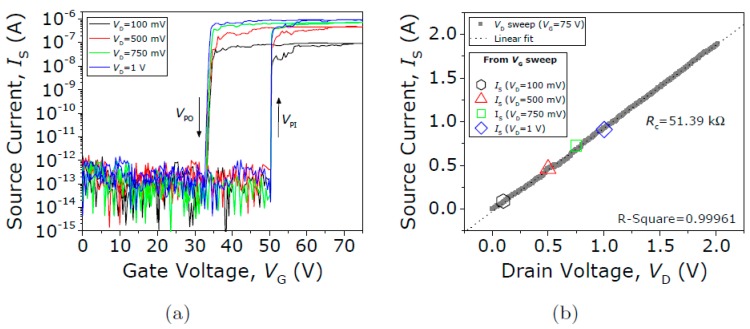
(**a**) Current voltage (*I-V*) curves for different V_D_ voltages. (**b**) I_DS_-V_DS_ sweep with the relay in its ON-state (V_G_ = 75 V), showing an ohmic dependence. The R_C_ values extracted from the *I-V* curves in (**a**) are also plotted (symbols).

**Figure 15:**
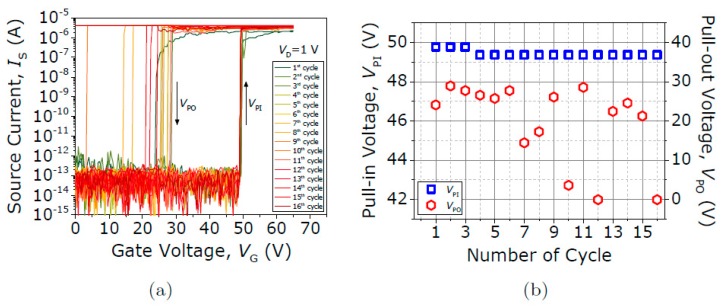
(**a**) Current voltage (*I-V*) curves for different V_D_ voltages. (**b**) Evolution of V_PI_ and V_PO_ over 16 cycles.

In contrast, the contact performance of the Type III seesaw relay is found to behave completely differently when it is operated under vacuum conditions. First, the initial native oxide breakdown is produced switching the device on (with V_GS_ = 75 V) and sweeping up the V_DS_ until the drain current spike is detected (see Figure 13). After this non-conductive oxide breakdown, I-V characteristics of the same relay for four different V_DS_ bias voltages are acquired (see Figure 14). The same pull-in and pull-out voltages are obtained no matter the V_DS_ bias used, as expected. Only the level of current in the ON-state is changed according with the V_DS_ bias. In Figure 14b, the R_C_ is computed sweeping the V_DS_ voltage while the relay is in its ON-state (V_GS_ = 75 V), obtaining a value of 51.4 kΩ. An attempt is then made to monitor the evolution of contact properties after each cycle by taking continuous I-V curves with fixed V_DS_ = 1 V (Figure 15). By doing so, *V_PI_* is found to be stable, but the relay is stuck after 16 cycles, which indicates a lower cycling life compared with the Type II seesaw relays. In Table 3, a brief summary of the three types of relays based on the seesaw torsional structure is provided.

## 4. Discussion and Conclusions

From the above characterization of the performance of the relays, we can state that a symmetric switching operation with a five-terminal torsional relay has been achieved, providing lower pull-in voltage and contact resistance (Type II, W/TiN contact) than previously reported, based on the same technological VIA3 platform [13] (see Table 3). In addition, if we compare the presented five-terminal torsional Seesaw device with relays already reported with TiN contacts, the seesaw relays provide the lowest contact resistance with higher cycling time. Only Reference [9] provides similar contact resistance, but they report lower switching life time and additionally these relays are not monolithically integrated into CMOS. The life time of the presented relay could be improved through the use of a proper vacuum packaging.

Overall, the five-terminal relay allows for the operation as two independent relays (left and right contact), with the guarantee that they will never be ON at the same time—one clear advantage over the CMOS transistor-based relays. This implies that a higher degree of compactness for mechanical digital logic circuits can be achieved. In this sense, the presented device is an advancement towards more robust and reliable mechanical relays which can provide a decrease in power consumption for portable and wearable devices.

## Figures and Tables

**Table 1:** Seesaw relay design parameters and their values.

Design Parameter	Value (µm)
Torsion beam length *L_T_*	4.7
Torsion beam width *W_T_*	0.5
Torsion beam thickness *T_T_*	1.3
Body length *L_B_*	59.6
Body width *W_B_*	16
Electrode length G_R,L_	30
Electrode width G_W_	16
Contact length *L_C_*	2.5
Contact width *W_C_*	2.5
Actuation gap *T_Gap_*	1.95
Contact gap (i) *T_con_*	1
Contact gap (ii) *T_con_*	1.3 ^a^
Contact gap (iii) *T_con_*	0.45 ^a^

^a^ The gap is measured after fabrication.

**Table 2:** CoventorWare simulation of the resonant frequencies and mode shapes of the seesaw relay.

	Torsional Mode	Vertical Mode
Mode Shape	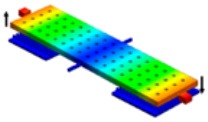	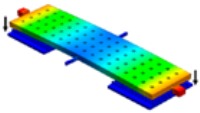
Resonant Frequency, *f*_0_ Spring Constant, *k_eff_*	152 kHz 1.28 N/m	1.5 MHz 69.6 N/m

**Table 3:** Summary of the characterized parameters of the torsional MEMS relays integrated monolithically using the VIA3 tungsten of the BEOL layer of the CMOS 0.35 µm technology.

Device	SEM Image Structural/Material	Contact Material	V_PI_ (V)	ON-OFF Ratio	R_C_ (Ω)	Cycles (EOL)
This work	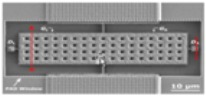 Via3 BEOL-CMOS layers	TiN/TiN	54.8 ^a^	10^5 a^	100 M ^a^	-
		W/TiN	57	10^7^	2 k	355
		W/W	49	10^7^	51.4 k	16
[13]	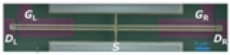 Via3 BEOL-CMOS layers	TiN/TiN	71.3	10^6^	20–45 k	65
[16]	CVD/PVD TiN, CMOS	TiN/TiN (annealed)	24	10^5^	500 M	30
[8]	TiN coated PolySiGe, no CMOS	TiN/SiGe	15	10^6^	No ohmic contact	10^10^
[17]	MIM module, CMOS	TiN/TiN	5–20	10^4^	1 G	10
[9]	TiN coated PolySi, no CMOS	TiN/TiN	22	>10^4^	4–15 k	150

^a^ Only measurements in air conditions due to the aluminum over-etching problem.
